# Clinical Value of Early Serum Albumin in Predicting Mortality Among Patients With Acute Pancreatitis: A Meta-Analysis

**DOI:** 10.7759/cureus.105664

**Published:** 2026-03-22

**Authors:** Mohamed A Helali, Saifalden Mustafa Badralden Mustafa, Mohamed Elhadi Mohamed Elbashir, Khalid Elwalid Ahmed Ali, Abubakr Humidan, Ammar Mohamed Ibrahim Obeidalla, Mohamed Mamoun Abbas Khalafalla, Bishoy Alfred William Dawis, Mohamed Shamseldin Mustafa Khalid, Alaa Kamal Hassan Hamedelnile, Mona Waheeb Khalifa Rahamt Alla, Mohamed Fatahelalim Ayed, Ahmad Mufid Kanaan, Maaz Mohamed Sharfeldin Nasreldin, Maher Abdallah Atieh Yousef, Dafaalla Salih

**Affiliations:** 1 Pediatric Surgery, Pediatric Surgery Center, National Ribat University Hospital, Khartoum, SDN; 2 Pediatrics, Atbara Teaching Hospital, Atbara, SDN; 3 Medicine, University of Gezira, Wad Madani, SDN; 4 Surgery, National Ribat University, Khartoum, SDN; 5 Surgery, London North West University Healthcare NHS Trust, London, GBR; 6 Surgery, Ibn Sina University, Khartoum, SDN; 7 Surgery, Elrazi University, Khartoum, SDN; 8 General Surgery, National Ribat University, Khartoum, SDN; 9 Surgery, University of Khartoum, Khartoum, SDN; 10 Surgery, Sudan Medical Specialization Board, Khartoum, SDN; 11 Surgery, Damascus University, Damascus, SYR; 12 Surgery, King Salman Hospital, Riyadh, SAU; 13 College of Medicine, Alzaiem Alazhari University, Khartoum North, SDN

**Keywords:** acute pancreatitis, low albumin, meta-analysis, mortality, systematic review

## Abstract

Acute pancreatitis (AP) is a critical illness that carries a considerable risk of severe complications and mortality. Serum albumin has been suggested as a marker of severity and worse outcomes in various critical illnesses. Accordingly, this meta-analysis was done to determine the clinical value of serum albumin in predicting mortality in patients with AP. A systematic review was conducted and reported in accordance with the Preferred Reporting Items for Systematic Reviews and Meta-Analyses (PRISMA) guidelines. The search was performed in PubMed, Scopus, and the WHO Virtual Health Library (VHL) to retrieve relevant studies assessing the association between serum albumin and mortality in patients with AP. Statistical analyses were done by calculating the pooled standardized mean difference (SMD) and area under the curve (AUC) estimates with 95% CI, using the meta package of R version 4.4.3 (R Foundation for Statistical Computing, Vienna, Austria). A total of 16 studies, including 8,394 patients, were included in the meta-analysis. The results indicated that non-survivors had significantly lower serum albumin levels than survivors (SMD = -0.79; 95% CI -1.11 to -0.47; p < 0.0001). Prognostic accuracy analysis demonstrated moderate discriminatory ability for serum albumin (AUC = 0.75; 95% CI 0.67 to 0.82; p < 0.001). This meta-analysis demonstrates that hypoalbuminemia is an important biomarker for identifying patients with AP who are at increased risk of mortality.

## Introduction and background

Acute pancreatitis (AP) is one of the leading causes of emergency hospitalizations [1]. The damage to pancreatic parenchyma activates a cascade of inflammation, causing a heterogeneous clinical course that ranges from mild and self-limited symptoms to life-threatening systemic complications, multiple organ failure, and death [2]. Despite advancements in critical care, mortality for severe cases remains high, often exceeding 20% in patients with severe disease [3]. Assessment of AP severity is crucial to predict the likelihood of a severe clinical course, which may involve organ failure and mortality [1]. Therefore, early risk stratification is essential to identify high-risk patients and initiate aggressive therapeutic interventions [4]. Early and accurate risk stratification not only guides clinical decision-making but also optimizes resource allocation and improves patient outcomes.

To this end, several widely used scoring systems exist, including the Bedside Index for Severity in Acute Pancreatitis (BISAP) score, Ranson criteria, and the Acute Physiology and Chronic Health Evaluation II (APACHE-II) score [3,4]. These tools incorporate a combination of clinical, physiological, and laboratory variables to estimate disease severity. While they demonstrate good predictive performance, their use in routine clinical practice may be limited by complexity, requirement for multiple parameters, or delayed applicability [1-5].

Serum albumin, the primary protein maintaining plasma oncotic pressure, plays a critical role in stabilizing the vascular endothelium and modulating systemic inflammatory responses [5]. In addition to its oncotic properties, albumin is involved in immune system regulation and the maintenance of normal capillary permeability and vascular integrity [5]. Hypoalbuminemia is frequently observed in AP, primarily driven by increased capillary permeability, which allows albumin to escape into the interstitial space [6]. This loss exacerbates fluid sequestration and tissue edema, potentially leading to hypovolemia and organ dysfunction. Therefore, low albumin levels may capture illness severity and predict adverse outcomes and mortality [6,7]. Previous studies have identified low serum albumin as a significant prognostic marker. For instance, Hong et al. demonstrated that hypoalbuminemia within 24 hours of admission is independently associated with persistent organ failure and death [7]. Similarly, a multicenter study by Ocskay et al. found that hypoalbuminemia affects approximately one-third of AP patients and dose-dependently increases mortality risk [6].

However, the results of previous studies regarding the consistency of albumin's predictive value have been inconclusive. In addition, no previously published meta-analyses have evaluated albumin as a prognostic biomarker for mortality in AP. Providing a more detailed comparison with existing literature helps highlight the novelty and added value of the present study. Furthermore, the interplay between hypoalbuminemia and other biochemical markers remains a subject of ongoing investigation. This review aimed to evaluate the association between on-admission hypoalbuminemia and in-hospital mortality in a diverse cohort of patients with AP, thereby reﬁning its utility as a clinical decision-making tool.

## Review

Methods

Search Approach

This review was conducted and reported based on the Preferred Reporting Items for Systematic Reviews and Meta-Analyses (PRISMA) guidelines [8] and was prospectively registered in the International Prospective Register of Systematic Reviews (PROSPERO) database (CRD420251135243). We conducted an inclusive search for all articles in three major databases - PubMed, Scopus, and the WHO Virtual Health Library (VHL) - up to November 2025. Search terms combined controlled vocabulary and free-text words for our keywords “acute pancreatitis”, “albumin”, and “mortality”, and the search was adapted for each database (Table 1).

**Table 1:** Search strategy VHL: Virtual Health Library

Database	Search terms
Pubmed	(("Pancreatitis" [tw] OR "pancreatic" [tw]) AND (("hypoalbuminemia" [tw] OR "albumin" [tw] OR "serum albumin" [tw] OR "Alb" [tw]) AND (("mortality" [tw] OR "death" [tw] OR "survival" [tw] OR "survivors" [tw] OR "fatality" [tw]))
Scopus	(TITLE-ABS-KEY ("Pancreatitis" OR "pancreatic") AND TITLE-ABS-KEY ("Pancreatitis" OR "pancreatic") AND TITLE-ABS-KEY ("mortality" OR "death" OR "survival" OR "survivors" OR "fatality"))
WHO VHL	Field 1 (Title, abstract, subject): ("Pancreatitis" OR "pancreatic") Operator: AND Field 1 (Title, abstract, subject): ("hypoalbuminemia" OR "albumin" OR "serum albumin" OR "Alb") Operator: AND Field 1 (Title, abstract, subject): ("mortality" OR "death" OR "survival" OR "survivors" OR "fatality")

Eligibility Criteria

Studies were considered eligible if they included adult patients with AP, reported serum albumin measured at admission or early during hospitalization, and evaluated in-hospital mortality as an outcome. Detailed inclusion and exclusion criteria are summarized in Table 2.

**Table 2:** Inclusion and exclusion criteria AUC: area under the curve; IQR: interquartile range; SMD: standardized mean difference; SD: standard deviation

Domain	Inclusion criteria	Exclusion criteria
Population	Adult patients diagnosed with acute pancreatitis, regardless of etiology	Pediatric populations, animal studies, or mixed populations without separable adult data
Exposure	Serum albumin measured at hospital admission or early during hospitalization	Studies without serum albumin measurement or measured only after major events or clinical interventions
Outcome	In-hospital mortality	Studies not reporting mortality or reporting only long-term outcomes without in-hospital mortality data
Study design	Observational studies reporting data allowing calculation of the effect size	Reviews, editorials, case reports, conference abstracts without sufficient data, and interventional trials without usable baseline observational data
Data requirements	Studies providing either mean ± SD or median (IQR) of serum albumin in survivors vs. non-survivors for SMD calculation, or studies providing AUC for serum albumin predicting mortality with 95% CI	Studies lacking extractable numerical data for SMD or AUC estimation

Data Extraction and Quality Assessment

An extraction form was used to collect the following data: bibliographic information (author, year, country), sample size, patient demographics, clinical data (severity classification of AP), serum albumin values in survivors and non-survivors, and area under the curve (AUC) values with 95% CIs when albumin was evaluated as a prognostic biomarker. The methodological quality and risk of bias were assessed using the Joanna Briggs Institute (JBI) critical appraisal checklist [9].

*Statistical* *Analyses*

Statistical analyses were carried out by calculating the pooled standardized mean difference (SMD) estimates with 95% CI to assess associations with serum albumin and mortality. Additionally, we assessed the predictive performance of serum albumin for predicting mortality, expressed as AUC. Pooled estimates of both SMD and AUC were calculated using the random-effects meta-analysis model. Between-study heterogeneity was quantified using the I^2^ statistic, and meta-regression analyses were performed to assess heterogeneity. Publication bias was explored by Egger’s regression test. All statistical analyses were conducted using the meta package of R version 4.4.3 (R Foundation for Statistical Computing, Vienna, Austria).

Results

Characteristics of the Included Studies

**Figure 1:**
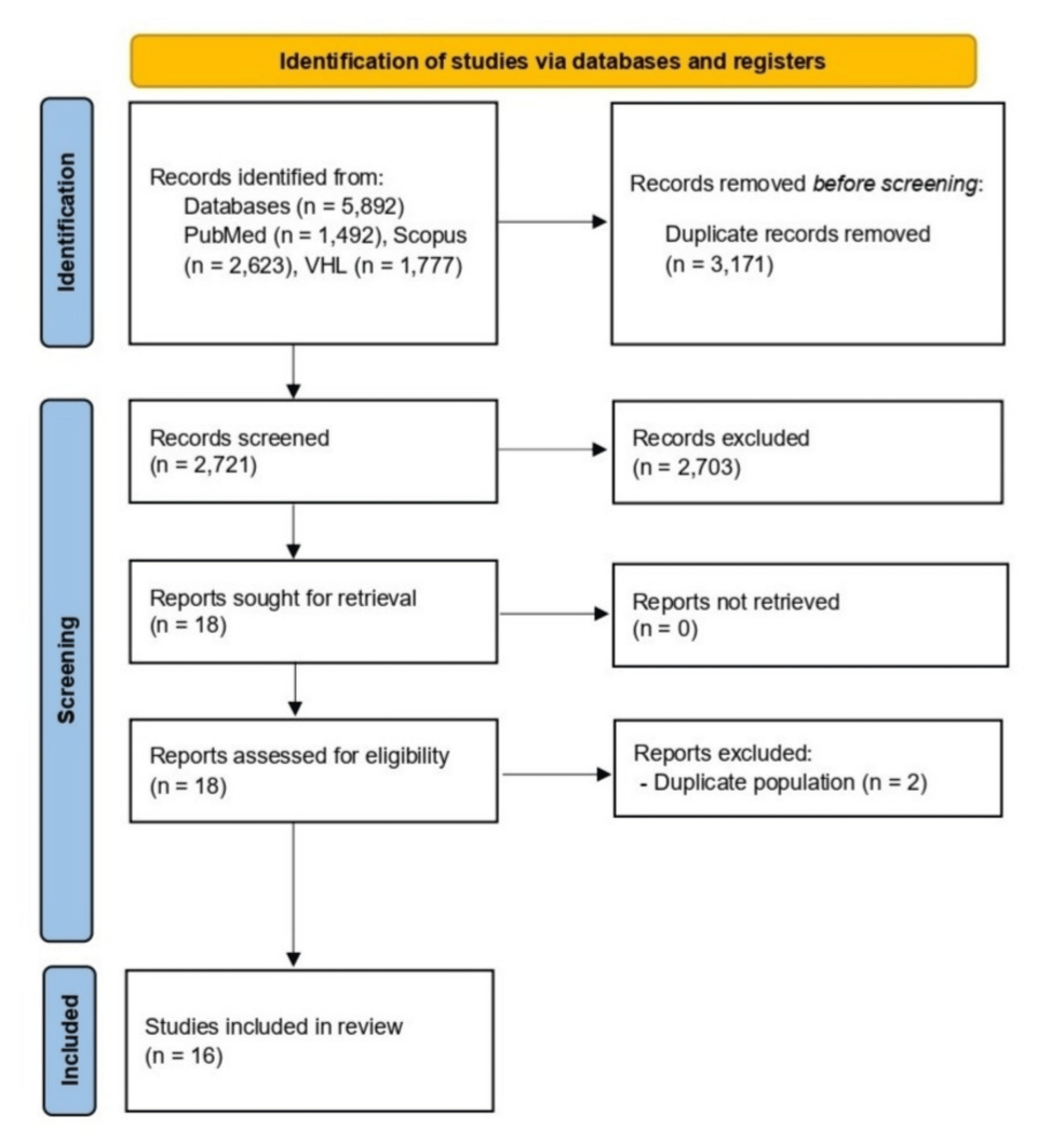
PRISMA flowchart of the study selection process PRISMA: Preferred Reporting Items for Systematic Reviews and Meta-Analyses; VHL: Virtual Health Library

The initial electronic database search yielded a total of 5,892 records. Following the removal of duplicate entries, 2,721 studies remained. These studies underwent a thorough title and abstract screening process. During this stage, 2,703 studies were excluded due to irrelevance, and full texts of the remaining records were screened (Figure 1). 

A total of 16 studies were included (Table 3) [6,7,10-23]. Studies were conducted across Asia, Europe, Africa, and North America, with a total sample size of 8,394 patients. The mean or median age across studies was generally in the fifth to sixth decade. Most studies focused on serum albumin levels directly as predictors of disease complications and mortality, while other studies assessed specific ratios, including the lactate-to-albumin ratio and the serum albumin-to-serum creatinine ratio, along with serum albumin. The quality assessment of the 16 included studies indicated a high level of methodological rigor using the 11-point JBI critical appraisal tool. Four studies fulfilled all assessment criteria, representing the highest tier of quality, and the majority of the research achieved a score of 10, demonstrating consistent robustness in study design and execution (Table 3).

**Table 3:** Summary of the characteristics of the included studies AP: acute pancreatitis; ACR: albumin-to-creatinine ratio; ED: emergency department; 1-SD: per one standard deviation; NA: not applicable

Study	Year	Country	No. of patients	Mean age (years)	Male (%)	Quality assessment	Albumin measurement	Summary of main findings
Amri et al. [10]	2024	Morocco	371	55.5 ± 18.6	29.1	10/11	Within 24 hours of admission (g/L)	Hypoalbuminemia was significantly associated with persistent systemic inflammatory response syndrome and mortality.
Du et al. [11]	2017	China	72	53.4 ± 11.2	73.6	10/11	Within 24 hours of admission (g/L)	Non-survivors had significantly lower serum albumin compared to survivors. Albumin was associated with in-hospital mortality on univariate analysis but not in multivariate analysis.
Efgan et al. [12]	2023	Turkey	457	56.28 ± 17.01	47.5	9/11	Upon ED admission (mg/dL)	Mean albumin level was significantly lower in high-risk AP, with moderate predictive ability for severe disease. In-hospital mortality was low, limiting assessment of albumin as an independent mortality predictor.
Güler and Ustaalioğlu [13]	2023	Turkey	634	59.7 ± 16.6	39.9	9/11	Upon ED admission (g/L)	Hypoalbuminemia was strongly associated with mortality; non-survivors had significantly lower albumin than survivors.
Han et al. [14]	2019	China	94	55.6 ± 18.3	56.4	10/11	Within 48 hours of admission (g/L)	Albumin levels were significantly lower in non-survivors. Low albumin was an independent predictor of mortality and strongly predicted death with an AUC of 0.866, sensitivity 70.4%, and specificity 92.5%. Combining low albumin with the Ranson score improved mortality prediction accuracy to 88.3%.
Hassan et al. [15]	2018	Egypt	129	53.6 ± 10.7	54.2	10/11	Upon ED admission (g/dL)	Mean albumin levels were significantly lower in non-survivors. Decreased albumin was an independent predictor of in-hospital AP mortality.
Hong et al. [7]	2017	China	700	48 (37-63)	59	11/11	Within 24 hours of admission (g/L)	Serum albumin was independently associated with mortality and persistent organ failure.
Liu et al. [16]	2022	United States	539	57 (54-71)	57.9	11/11	Upon ED admission (g/L)	Non-survivors had significantly lower serum albumin levels than survivors. Albumin alone and the lactate-to-albumin ratio showed moderate prognostic value for mortality.
Ni et al. [17]	2022	China	199	52.33	NA	9/11	Within first 72 hours of admission (g/L)	Albumin and prealbumin showed moderate prognostic value for mortality in patients with severe AP.
Ocskay et al. [6]	2021	Hungary	1,149	NA	NA	11/11	Within first 48 hours of admission (g/L)	Hypoalbuminemia dose-dependently increased the risk of mortality, local complications, longer hospital stay, and organ failure.
Qin et al. [18]	2024	China	240	49.23 ±14.21	59.17	10/11	Within 24 hours of admission (g/L)	Hypoalbuminemia was strongly associated with the severity of AP and mortality in patients with severe AP.
Selvanathan et al. [19]	2022	India	68	39.9 ± 11.8	95.6	9/11	Within 24 hours of presentation (mg/dL)	Serum albumin levels were significantly lower in non-survivors compared to survivors. Hypoalbuminemia at presentation was associated with increased in-hospital mortality.
Yang et al. [20]	2024	China	114	59.6 ± 16.1	46.5	10/11	Upon ED admission (g/L)	Lower serum albumin and ACR were independently associated with higher in-hospital mortality in patients with severe AP. Patients in the low ACR group (≤0.206) had significant mortality (62.3%) compared to the high group (18.0%).
Zhang et al. [21]	2017	China	166	53.7 (24-79)	62.7	11/11	Within 24 hours after admission (g/L)	Lower albumin levels were independently associated with higher mortality. Non-survivors had significantly lower albumin compared to survivors. With a cut-off at 34.95 g/L, sensitivity was 72.2% and specificity was 97.3%.
Zhao et al. [22]	2023	China	248	59.5 (39-70)	56.69	10/11	Within 24 hours after admission (g/L)	Lower albumin levels were associated with the severity of AP, mortality, organ failure, and pancreatic necrosis.
Zou et al. [23]	2026	China	3,214	49.2 ± 13.9	62.8	10/11	Within 24 hours after admission (g/L)	Patients with the lowest cumulative albumin exposure tertile had significantly higher mortality (11.0%) compared to those in the highest tertile (1.3%). Cumulative albumin exposure showed a dose-response inverse association with in-hospital mortality, with each 1-SD increase associated with a 42% reduction in mortality risk.

Serum Albumin Levels Among Survivors vs. Non-survivors 

**Figure 2:**
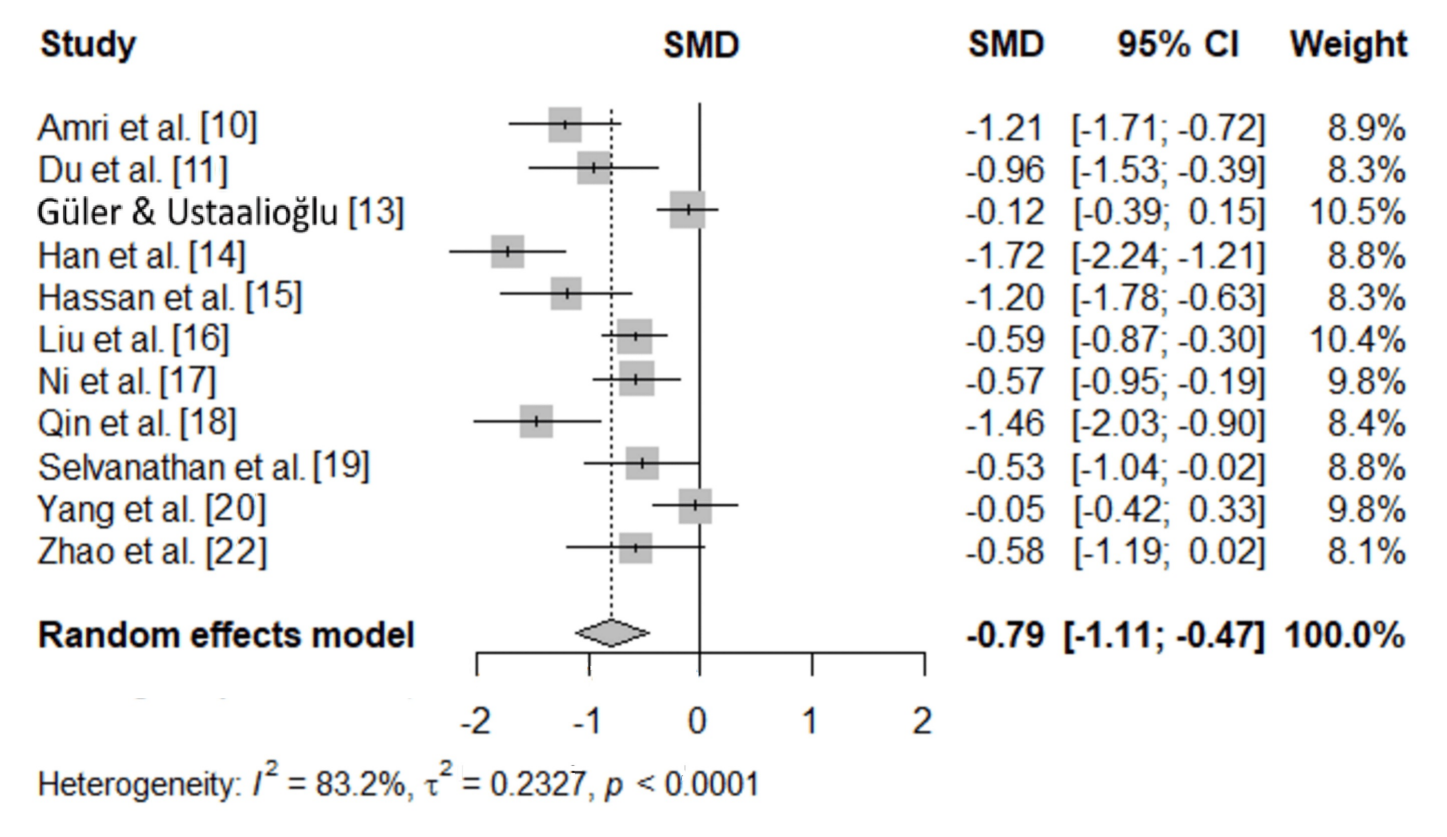
Forest plot of pooled SMD estimates for serum albumin in survivors vs. non-survivors SMD: standardized mean difference

A total of 11 studies, with varied sample sizes and clinical settings, were included in the quantitative analysis. The meta-analysis demonstrated that serum albumin levels were significantly lower in non-survivors compared to survivors, with an SMD of -0.79 (95% CI: -1.11 to -0.47, p < 0.0001), indicating a moderate to large effect size (Figure 2).

Between-study heterogeneity was detected (I^2^ = 83.2%), reflecting variability in study characteristics and populations. Despite this heterogeneity, the overall direction of effect consistently favored lower albumin levels among non-survivors. Egger’s regression testing of funnel plot asymmetry was significant (p = 0.018). The meta-regression results showed that the magnitude of association between serum albumin and mortality was not affected by any of the characteristics of the included studies, such as age (coefficient = 0.01, p = 0.392), proportion of male patients (coefficient = -0.0004, p = 0.965), and sample size (coefficient = -0.0009, p = 0.263).

Leave-one-out sensitivity analysis confirmed the robustness of the findings. It showed that excluding individual studies yielded pooled SMDs ranging between -0.69 and -0.86, with all associations remaining statistically significant (p < 0.0001), indicating that no single study excessively influenced the overall result.

Diagnostic Performance Results

**Figure 3:**
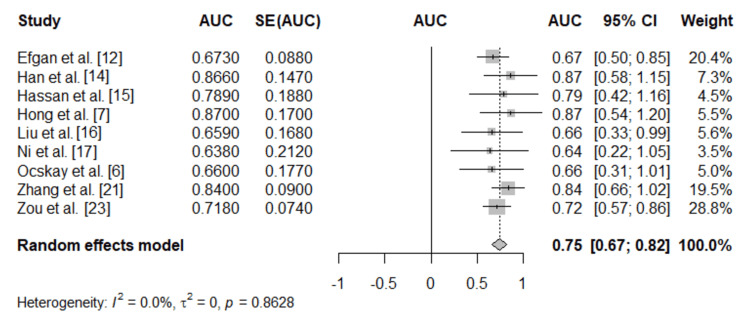
Forest plot of pooled AUC estimates for serum albumin predicting mortality AUC: area under the curve; SE: standard error

A total of nine studies evaluated the prognostic value of serum albumin for mortality, with performance expressed as AUC. The pooled random effects estimate demonstrated that serum albumin had moderate discriminatory ability, with a summary AUC of 0.75 (95% CI: 0.67 to 0.82, p < 0.001) (Figure 3), indicating that lower serum albumin levels are consistently associated with higher mortality risk in AP. Heterogeneity was not observed among the included studies (I^2^ = 0.0%). Publication bias analysis using Egger’s regression test did not indicate the presence of small-study effects (p = 0.898).

To examine the robustness of the findings, leave-one-out sensitivity analysis was performed. The sequential omission of each study did not substantially change the pooled effect, with summary AUC values ranging between 0.73 and 0.76, indicating that no single study disproportionately influenced the overall results. Furthermore, the meta-regression results showed that the effect size was not affected by the albumin cut-off thresholds applied by the included studies (coefficient = -0.007, p = 0.089), indicating that prognostic performance was consistent across different albumin thresholds used by the studies. 

Discussion

This systematic review and meta-analysis provided evidence that serum albumin levels are significantly lower in AP non-survivors, with a moderate discriminatory ability for mortality prediction. The main findings remained robust across different sensitivity analyses.

These findings are consistent with pathophysiological mechanisms that may explain AP-related hypoalbuminemia, such as the excessive release of inflammatory cytokines like IL-1, IL-6, and TNF-α in AP, which can inhibit hepatic albumin synthesis [7,10]. The resultant hypoalbuminemia represents the loss of albumin's important functional properties, including antioxidant, anti-inflammatory, and endothelial protective functions, which account for a large proportion of plasma antioxidant activity [24]. Additionally, these findings are consistent with results regarding the impact of hypoalbuminemia in other diseases with a severe inflammatory response, such as severe sepsis and septic shock [6].

The predictive value found for serum albumin was comparable to other complex scoring systems that incorporate multiple clinical and laboratory variables, such as APACHE-II, BISAP, and Ranson scores. Moreover, serum albumin carries additional advantages, such as being a single and readily available parameter that can be measured on admission, as well as having a lower cost than complex scoring systems [25]. Furthermore, Han et al. showed that combining low albumin with the Ranson score improved mortality prediction accuracy to 88.3%, suggesting that albumin can enhance existing prognostic models [14]. Importantly, our findings indicate that serum albumin demonstrates only moderate predictive performance for mortality, suggesting that it could be a useful complementary biomarker alongside other scores like APACHE-II, BISAP, and Ranson scores, which typically achieve higher predictive accuracy with AUC values frequently ranging from approximately 0.80 to >0.90 for mortality and severe disease prediction [26]. The reviewed studies showed that combining serum albumin with established scoring systems enhances prognostic performance.

A further dose-dependent relationship between albumin levels and clinical outcomes has been demonstrated by some of the included studies [6,23]. Ocskay et al. in a large multicenter cohort of 1,149 patients showed that this dose-dependent relationship extends to other adverse outcomes, including organ failure, local complications, and length of hospital stay [6]. This dose-response pattern strengthens the causal inference regarding the relationship between hypoalbuminemia and adverse outcomes.

The practical implementation of albumin-based risk stratification should include routine measurement of serum albumin in the admission workup, with serial monitoring particularly important in patients with worsening clinical status. Furthermore, several studies in this meta-analysis have explored novel albumin-based composite biomarkers that may enhance prognostic accuracy. Although this meta-analysis clearly establishes hypoalbuminemia as a prognostic marker, the therapeutic value of albumin replacement treatment is controversial and requires further investigations and careful consideration.

The heterogeneity observed in this meta-analysis likely reflects differences in study populations, geographic variation, varying severity distributions, and different AP etiologies. However, several factors support the robustness of our findings despite this heterogeneity. First, the direction of effect was consistent across all studies, with lower albumin levels uniformly associated with worse outcomes. Second, meta-regression analyses demonstrated that the magnitude of association was not significantly affected by major study characteristics, including age, sex proportion, sample size, or albumin cut-off thresholds. Third, leave-one-out sensitivity analyses confirmed that no single study disproportionately influenced the overall results, with pooled estimates remaining stable and statistically significant regardless of which study was excluded.

This meta-analysis has some limitations that should be acknowledged. The high heterogeneity between studies, as explored through meta-regression and sensitivity analyses, reflects inherent differences in populations that could not be fully accounted for. Publication bias was detected in the SMD analysis, suggesting that smaller studies with null or negative findings may be underrepresented in the literature. Future research should focus on prospective validation studies to establish optimal cut-off values for clinical decision-making.

## Conclusions

This meta-analysis demonstrates that hypoalbuminemia is an important biomarker for identifying patients at increased risk of mortality among those presenting with AP. The results indicated that non-survivors had significantly lower serum albumin levels than survivors (SMD = -0.79; 95% CI -1.11 to -0.47; p < 0.0001). Prognostic accuracy analysis demonstrated moderate discriminatory ability for serum albumin (AUC = 0.75; 95% CI 0.67 to 0.82; p < 0.001). The results refined the utility of serum albumin as a clinical decision-making tool. Given its affordability and accessibility, hypoalbuminemia should be routinely incorporated into clinical practice to facilitate the timely identification of high-risk patients and improve patient outcomes.
